# Supplementary material for: π-phase modulated monolayer supercritical lens
Source: Nat Commun. 2021 Jan 4;12:32. doi: 10.1038/s41467-020-20278-x (PMC7782844; doi:10.1038/s41467-020-20278-x)
Supplement: Supplementary file 1 — Supplementary Information [file 41467_2020_20278_MOESM1_ESM.pdf]

Supplementary Information for

π-phase modulated monolayer supercritical lens

Fei Qin[1#], Boqing Liu[2#], Linwei Zhu[3], Jian Lei[1], Wei Fang[1], Dejiao Hu[1], Yi Zhu[2],

Wendi Ma[2], Bowen Wang[2], Tan Shi[1], Yaoyu Cao[1], Bai-ou Guan[1], Cheng-wei Qiu[4],

Yuerui Lu[2,5,6], Xiangping Li[1*]*

[1]Guangdong Provincial Key Laboratory of Optical Fiber Sensing and Communications, Institute of Photonics Technology, Jinan University, Guangzhou, 510632, China

[2]Research School of Electrical, Energy and Materials Engineering, College of Engineering and Computer Science, the Australian National University, Canberra, ACT 2601, Australia

[3]School of Physics and Optoelectronic Engineering, Ludong University, Yantai, 264025, China

[4]Department of Electrical and Computer Engineering, National University of Singapore, 4 Engineering Drive 3, Singapore 117583, Singapore

[5]ARC Centre of Excellence in Future Low-Energy Electronics Technologies (FLEET), ANU node, Canberra, ACT 2601, Australia

[6]Centre for Quantum Computation and Communication Technology, Department of Quantum Science, Research School of Physics and Engineering, The Australian National University, Acton, ACT 2601, Australia

[#] These authors contributed equally to this work

**Corresponding author: xiangpingli@jnu.edu.cn, yuerui.lu@anu.edu.au*

This file includes:

Supplementary Note 1.
Optimization of the compound system parameters by Transfer Matrix Methods

Supplementary Note 2.
Preparation and characterization of atomic thin MoS_2 on well-designed substrate

Supplementary Note 3.
Design of binary phase supercritical lens on MoS_2 sheet

Supplementary Note 4.
Dependence of focusing properties on the lens parameters

Supplementary Note 5.
Analysis of the focusing efficiency of the atomic thin supercritical lens

Supplementary Note 6.
Demonstration of multifunctional capability by broadband grating deflection and holography.

Supplementary Table 1.
Parameters of supercritical lens for sub-diffraction limit focusing.

Supplementary Figures 1-19

Supplementary Note 1.

Optimization of the compound system parameters by Transfer Matrix Methods:

Lying a monolayer MoS_2 sheet on a uniform substrate separated by a dielectric insulator layer properly, the abrupt Heaviside shift in the reflection phase can be readily achieved. Considering the compound system into a single port four layers system with Air-MoS_2-ZnO-Si, the complex reflection coefficient from the MoS_2 sheet and dielectric layer can be simulated by using the transfer-matrix methods, and the phase shift for the system with ($\emptyset_{\mathrm{MoS_2}}$) and without MoS_2 sheet ($\emptyset_{\mathrm{subs}}$), can be precisely calculated. The transfer matrix for a multilayer system containing m+1 layer can be described as follows:

$$M = \begin{bmatrix} A & B \\ C & D \end{bmatrix} = M_{\mathrm{m-1,m}} P_{\mathrm{m-1}} M_{\mathrm{m-2,m-1}} \dots \dots M_{12} P_1 M_{01} \qquad (1)$$

Where P_i denotes the transfer matrix of propagation within layer i, and $M_{\mathrm{i,j}}$ represents the transfer matrix from layer i to j[1]. By converting the transfer matrix into scattering matrix, the complex reflection for m+1 layer system can be expressed as:

$$r_{\mathrm{0,m}} = -{}^{C}/_{D} \qquad (2)$$

Then the monolayer MoS_2 sheet induced phase difference $\Delta\Phi$ as the function of refractive index (n) and the thickness (t) can be readily derived.

For the optimal parameters, the phaser diagram in Fig. 1d is calculated by sweeping the thickness of the ZnO buffer layer. Here, the interest wavelength of 535 nm is coming from the fixed wavelength (535 nm) of our phase-shifting interferometry (PSI) that is used to verify the phase delay in experiments[2]. The refractive indices we used are $n_{\mathrm{air}} = 1$; $n_{\mathrm{dielectric}} = 1.98$; $\tilde{n}_{\mathrm{MoS_2}}$ and $\tilde{n}_{\mathrm{si}}$,, where the $\tilde{n}_{\mathrm{MoS2}}$ and $\tilde{n}_{\mathrm{si}}$ taken from ref [3] and ref [4]. As shown in Fig. 1d, trajectories of complex reflection coefficients fall into different quadrants of the complex plane (representing under- and over- coupling states), which manifest that distinct reflection phases are mainly determined by both the absorption loss and the radiation loss of the compound structure. By coating and removing the monolayer MoS_2 film from the substrate, the largest swing of the reflection phase difference of π can be achieved through finely parametric tuning of the dielectric layer in terms of refractive index and thickness.

As indicated from Eq.1 in main text, the abrupt phase shift arising from the system state transition between under-coupling and over-coupling can be obtained by tuning the ratio of Q_r /Q_a. Considering the Air-MoS_2-ZnO-Si system, the Q_a related to absorption rate is subject to the thickness of the lossy MoS_2 sheet. Since only invariable monolayer MoS_2 sheet is applied in our configuration, the Q_a will be a constant in a certain region. However, the Q_r related to the radiation rate is readily to be modified by changing the thickness of the dielectric layer. We calculated the complex reflection coefficient of the system with different parameter combinations along the state transition trajectory (white dash curve in Supplementary Fig. 1a) by using the transfer matrix method (TMM), and obtained the of Q_r and Q_a values by fitting with Eq. 1 to the calculation results. Supplementary Fig. 1b shows the evolution of fitted Q_r and Q_a as a function of thickness and refractive index of the dielectric layer. The results indicate that the absorption parameters Q_a remain nearly constant value of 1.03. But the radiation parameters Q_r gradually increases from 0.95 to 1.15 while the refractive index increases from 1.8 to 2.0. The nontrivial phase shift occurs at the critical parameter of n = 1.89 where Q_r /Q_a = 1, corresponding to the critical coupling point as marked by the black star in Supplementary Fig. 1. Consequently, as shown in Supplementary Fig. 1, monolayer MoS_2 on the dielectric layer with refractive index n = 1.8~2.0 and thickness t = 65~66 nm could support a giant phase difference to the level of π. Supplementary Fig. 2 illustrates an exemplary case of under-coupling states with fitting results of Q_a = 1.03 and Q_r = 1.12, in which the refractive index of dielectric layer n = 1.98 and thickness t = 65 nm.

To reveal the role of losses *(k* value of the MoS_2 film) and validate the loss-assisted phase modulation principle, we fixed all the other parameters and swept the imaginary part (k) of MoS_2 film in the TMM simulation. As shown in Supplementary Fig. 14, the critical coupling point for state transition between under-coupling and over-coupling, as well as the π phase condition, will be shifted to longer wavelengths along the decreasing of the imaginary part (k) of refractive index. Fortunately, the refractive index of MoS_2 in the wavelength range of 450-580 nm also changes with the same trend. Thus, it holds the key for the broadband response.

Supplementary Note 2.

Preparation and characterization of atomic thin MoS_2 on well-designed substrate

Dielectric materials, Al_2O_3 and ZnO, were deposited on the doped silicon wafers by using Atomic Layer Deposition (ALD) system (Cambridge Nanotech ALD Fiji F200). Mono-layer and bi-layer MoS_2 samples were prepared by mechanically exfoliation and then transferred onto SiO_2/Si chips. The layer number of MoS_2 samples was identified by optical contrast and Raman spectroscopy[5]. After the exfoliated MoS_2 samples were transferred onto the ZnO/Si and Al_2O_3/Si substrates, optical path length was measured to derive the phase shift difference between MoS_2 sheets with substrate (Fig. 2 and Supplementary Fig. 5). All the optical path length (OPL) and phase shift characterizations of processed samples were measured under 535 nm light source by using a Veeco NT9100 phase-shifting interferometer (PSI) in the ambient environment[2]. Measured phase shift of the MoS_2 flake on the ZnO substrate was defined by $\Delta\emptyset = \emptyset_{MoS_2} - \emptyset_{ZnO}$, where $\emptyset_{MoS_2}$ and $\emptyset_{ZnO}$ are the PSI measured phase shifts of the light reflected from the MoS_2 flake and the ZnO substrate, as depicted in Fig. 1a, respectively.

Raman characterization was performed to confirm the fidelity of MoS_2 sheet by using Renishaw's inVia Raman Microscope. A 532 nm diode-pumped solid state (DPSS) laser (Renishaw RL532C50) was used as the excitation source for the Raman measurement. The laser power was maintained at the same level and the testing environment is in the ambient environment. As shown in Supplementary Fig. 3, both the mono- and bi-layer samples have A_{1g} and E^1_{2g} modes. However, the Raman shift differences between two modes of different layers will increase with the layer number. The distances between two peaks, 19.5±0.3cm^{-1}, 22.4.5±0.3cm^{-1}, and 24.6 ± 0.2 cm^{-1}, can be used to confirm transferred samples are monolayer, bilayer and bulk MoS_2 samples, respectively. In the Raman spectra, all modes and frequencies of MoS_2 samples and substrates are clearly shown, except the ZnO. This is because the ALD grown ZnO film could not have well crystallinity until the thickness is over certain value (~100 nm) and the preferential growth in the c-axis dominates. Therefore, the Raman modes of ZnO are not distinguishable[6].

The CVD grown chip-scale MoS_2 samples were synthesized on SiO_2/Si substrates in a hot-wall furnace. Prior to the growth, PTCDA solution was used to treat the substrate. High purity MoO_3 and S powder (Sigma Aldrich) were placed in two separate quartz boats and the substrates were faced down and placed on the upper side of MoO_3 power. The MoS_2 samples were fabricated by annealing at 630 ℃ for 10 - 20 minutes with a heating rate of 15 ℃ /min and N_2 or Ag flow (1 sccm) at ambient conditions. Wet transfer method was used to transfer the CVD-grown mono- and bi-layer MoS_2 samples onto ZnO/Si substrates for lens making. After CVD growth, a polystyrene polymer thin film layer was deposited on top of MoS_2/SiO_2/Si substrate, using spin coating at 3000 rpm for 60s, followed by a baking process at 90 °C for 30 min. Then the chip was soaked into DI water, the polystyrene polymer film carrying MoS_2 samples peeled off from SiO_2/Si substrate and floated on water after around 2 hours. ZnO/Si substrate was used to lift up the MoS_2 polymer film stack, followed by a baking process at 100 °C for 1 hour. At last, we soaked the chip into toluene solution for 24 hours to fully remove polymer film, ensuing no organic residues would exist on top of MoS_2 layer.

Supplementary Note 3.

Design of binary phase supercritical lens on MoS_2 sheet:

The design principle of SCL is based on the Rayleigh-Somerfield diffraction theory combined with the particle swarm optimization algorithm (PSO), which have been reported in our previous works[7, 8]. The diameters of the lens as well as the numerical apertures are set on basis of the customized characterization system, as shown in Supplementary Fig. 8. The radius of outermost belts is 20 μm, and the numerical aperture is set in $NA = 0.4$. To facilitate the fabrication process by laser scribing, we set all the scribed belts with a fixed width of 500 nm. Therefore, we only need to optimize the central radius of each belts by using the particle swarm algorithm to get a sub-diffraction limited lateral spot size and high uniform optical needle. By tuning the central radius of each belts in the optimization process, a phase type SCL with $NA = 0.4$ has been created at the wavelength of 535 nm, and sub-diffraction limited focusing properties in the far field can be achieved by the constructive interference between 0-phase in scribed regions and π-phase in un-scribed regions. For achieving broadband response of the sub-diffraction limited focusing, we have

improved the optimization algorithm and established a multi-channel optimization algorithm mechanism, which including two step evaluations as shown in Supplementary Fig. 15: (I) Generate the position and width data set of belts position randomly by the particle swarm optimization algorithm, then taking the set of parameters to form corresponding binary phase supercritical lens at the wavelength of 535 nm, and evaluate the focal performance of lenses such as the intensity (I(z=f))of the focus, the lateral size (FWHM) of the focus, and the contrast (Iz/I(z=f)) at the focal plane. (II) If the target value for 535 nm is satisfied, then evaluate the focal performance of lenses at the relative focal plane for wavelength 435 nm and 585 nm. Compare the obtained evaluation function value with the final target value, if the target value's precision is reached, the optimization is completed and the optimization parameter is output. Otherwise, enter the next generation until the precision of the target value is reached. Since the optimization target of supercritical focusing at 535nm was set as 0.42λ/NA, it has considerable structural tolerance to allow the broadband focusing performance than that of the optical super-oscillation (<0.38λ/NA). Thus, one could find a set of optimal structure parameters to satisfy the targets for wavelengths of 435nm and 585nm simultaneously. When the output pattern from optimization could satisfy the sub-diffraction limited focusing requirement for the wavelength 435 nm, 535 nm, and 585 nm simultaneously, the set of parameters would be the applicable design for broadband supercritical lens. The parameters of optimized lens are shown in Supplementary Table 1. The target focal spot size has a radius of 0.42λ/NA which in between the Rayleigh Criterion (RC, 0.61λ/NA) and Super-Oscillation Criterion (SOC, 0.38λ/NA)[7].

Supplementary Note 4.

Dependence of focusing properties on the lens parameters

As we expressed in the lens design part, the supercritical lens composes of a series concentric laser scribed binary belts on the monolayer MoS_2 sheet. To facilitate the fabrication process, only the central radius of each belts is determined during the optimization process, the phase depth as well as the width for all belts are fixed. However, all these parameters could be slightly deviated from the ideal conditions, and will definitely influence the focusing performance experimentally. As shown in Supplementary Fig. 9,

while the phase shift depth of MoS_2 sheet changes from π to 0.2π with other parameters fixed at the same values as those used in Fig. 3, the focusing effect becomes weaker because of the insufficient light modulation. The focal length and lateral size of the focal spot remain intact, but the intensity distribution in the optical needle deviates from the simulation along with the decrease in phase modulation strengths, which can explain the gently deviation of the measured optical needle with the simulation one between Figs. 3g and 3h. In addition, the width of each scribed belts also affects the focusing properties as well. As discussed in previous works, each belt provides a spatial frequency component in the simulation process by vectorial Rayleigh-Sommerfeld technique. Suppose that the belts are infinite narrow, it will act like a zero-order Bessel function in the focal plane[9]. However, all the actual belts have certain widths which makes the spatial frequency components have a bandwidth. Such impure light field components lead a bias for the focal spot size, as shown in Supplementary Fig. 10. The lateral size will be shrunk with the decreased belt width, but the focal spot intensity also decreases which lowers down the energy efficiency. Taking the conditions of lateral size, intensity, and the capability of laser scribing facility into consideration, belts width w = 0.5 μm is selected in the lens fabrication.

Supplementary Note 5.

Analysis of the focusing efficiency of the atomic thin supercritical lens

In this work, the focusing efficiency η is defined as the ratio between the energy concentrated in the sub-diffraction limited focal spot region and the direct reflection from the lens plane. The light intensity can be calculated by using the follows equation:

$$I(\rho) = \iint_{\Omega} f(x,y)\, d\sigma = K\left(\int_0^{2\pi}\int_0^{R} c(\rho)\rho d\rho d\varphi\right) \quad (3)$$

where Ω is the integration region, f indicates the light intensity as a function of the integral position, $c(\rho)$ is the light intensity on the optical detector, ρ is the radial coordinate at the integral plane, and R is the external radius of the integral region, φ is the rotation angle, K is a constant coefficient which is related to the system configuration and the integration time of the optical detector during the field intensity capturing. The focusing efficiency η can be defined as the ratio between the integration energy at the focal spot region (E_F) and the direct reflection energy at the lens region (E_L), as described as:

$$\eta = \frac{E_F}{E_L} = \frac{\int_0^{2\pi}\int_0^{r_F} I(z=f,\rho)\rho d\rho d\varphi}{\int_0^{2\pi}\int_0^{R_L} I(z=0,\rho)\rho d\rho d\varphi} \tag{4}$$

where f is the focal length, R_L is the outer radius of the supercritical lens, r_F is the radius of the mainlobes of the sub-diffraction limited focal spot as shown in Supplementary Fig. 12. Subsequently, by numerically integrating the light intensity over the lens plane and focal spot region, the experimental measured focusing efficiency for the optimized wavelength 535 nm around 1.2% is obtained in our work, which is similar with previously reported sub-diffraction limited metalens[10, 11].

Supplementary Note 6.

Demonstration of multifunctional capability by broadband grating deflection and metaholograms.

To further corroborate the phase modulation capability of the atomic thin MoS_2 sheet, a grating with 2μm pitch was patterned on the bilayer MoS_2 sheet via fs laser direct scribing system. By controlling the laser processing parameters, the MoS_2 nanoribbons of 1μm could be properly fabricated, and forming a grating with 50% aspect ratio. Supplementary Figs. 18a-18b) shows the optical microscopy image and its sectional SEM view of the scribed atomic thin grating. A super-continuum laser (Fianium WL-SC-400-4-PP) is used as the light source for illumination. As Supplementary Fig. 18c shown, beam deflection could be clearly observed in the spectrum region from Blue to Yellow color.

We also demonstrated an atomically thin meta-holograms by pixelated binary patterns on bilayer MoS_2 sheet, as presented in Supplementary Fig. 19 for monochromatic holographic results. A logo of Jinan University (Supplementary Fig. 19a) is used with permission as the original image for the holographic demonstration. The binary phase pattern for holographic images generation was obtained through a computer-generated hologram, then the fs laser direct scribing system was applied for the pattern fabrication. The pitch of the pixelated pattern was set to 1μm, and the overall size of meta-hologram pattern is 1000 μm × 1000 μm. The key parameters of fs laser we used in the fabrication process are as follows: repetition rate of 1 kHz, pulse width of 40 fs, and central wavelength of 800 nm. An objective lens with NA = 0.75 (Leica N Plan 50X/0.75) is used in the laser scribing system to make sure the spot size in laser scribing process is slightly smaller than

the pitch size 1μm. The optical image of the scribed meta-optics pixel arrays and zoom-in view SEM image are shown in Supplementary Figs. 19b-19c). Three lasers with wavelengths of 473 nm (MBL-III-473nm, CNI laser), 561 nm (MGL-FN-561nm, CNI laser) and 633 nm (DH-HN250P, Daheng Optics) were used for the optical characterizations. By using a customized characterization system, the reconstruction holographic images at the wavelength of 473 nm, 561 nm and 633 nm can be clearly obtained, as shown in Supplementary Figs. 19d-19f. Combining with the demonstration of supercritical lens, meta-grating, and meta-holographic images, the phase modulation capability of atomic thin MoS_2 are comprehensively validated.

Supplementary Table 1 Parameters of the supercritical lens for sub-diffraction limit focusing. R_n indicates the central radius of each scribed belts, and all the belts with a fixed width of 500 nm

**Belts Nos.**	**Radius R_n (μm)**	**Belts Nos.**	**Radius R_n (μm)**
**1**	0.75	**9**	11.55
**2**	2.15	**10**	12.85
**3**	3.25	**11**	14.35
**4**	4.95	**12**	16.15
**5**	6.35	**13**	17.55
**6**	7.65	**14**	19.05
**7**	9.15	**15**	20.35
**8**	10.35		

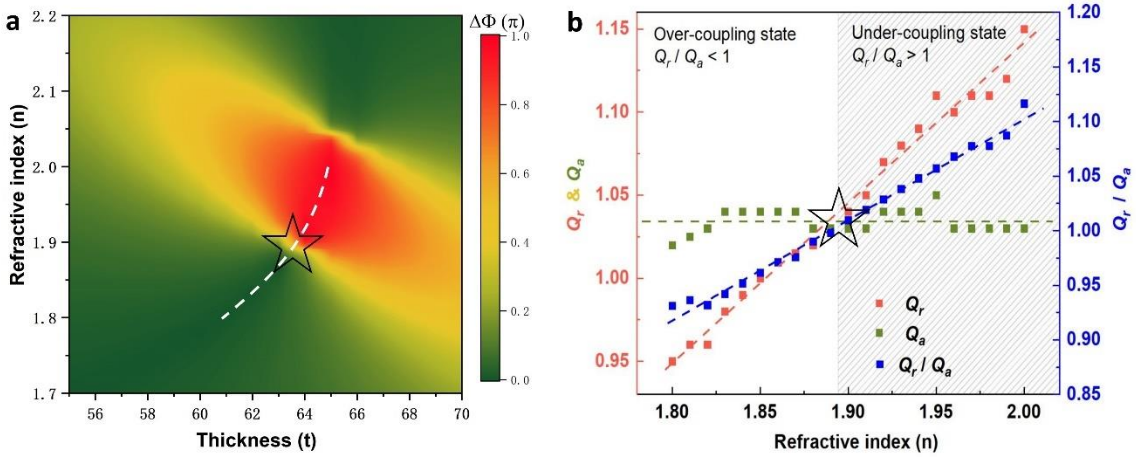

Supplementary Figure 1. Coupled-mode theory fitting results for the reflection results obtained by transfer matrix method for monolayer MoS_2. (a) Simulation results of loss-assisted phase modulation dependence on the thickness (t) and refractive index (n) of the dielectric layer. The white dashed line is the evolution trajectory of system states. The black star region represents the critical coupling point where the system transfers from over-coupling to under-coupling states, then leads to an abrupt phase change of the reflected light. (b) The evolution of the fitted parameters Q_r and Q_a along the dashed line in (a). The left part with $Q_r/Q_a < 1$ denotes the over-coupling state, and the shadow region in the right part with $Q_r/Q_a > 1$ denotes the under-coupling. The black star marks the critical coupling point region with $Q_r/Q_a \approx 1$.

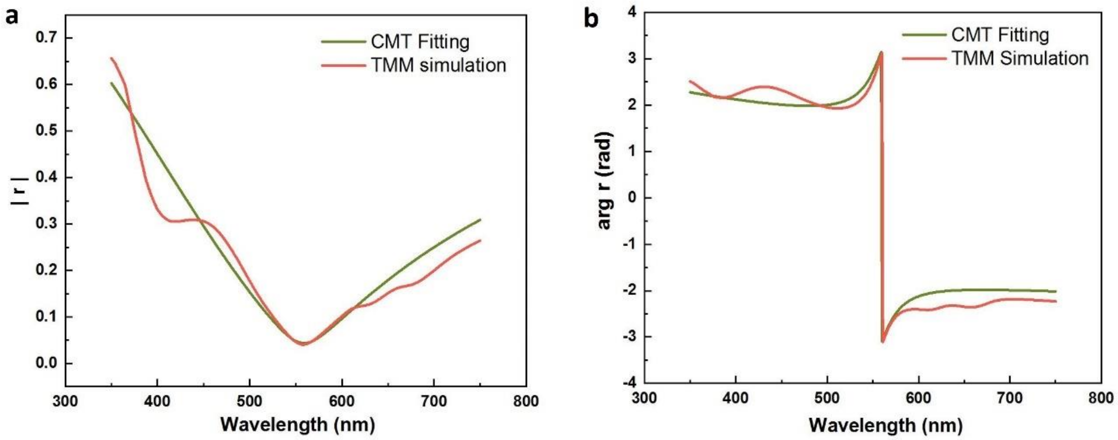

Supplementary Figure 2. Reflection coefficients from MoS_2-ZnO-Si system calculated by TMM (olive green lines) and fitted by CMT (orange line). The modules and phase of the coefficients are plotted on (a) and (b). The refractive index *n* and thickness *t* of the dielectric layer we used in here are *n* = 1.98, and *t* = 65 nm, respectively.

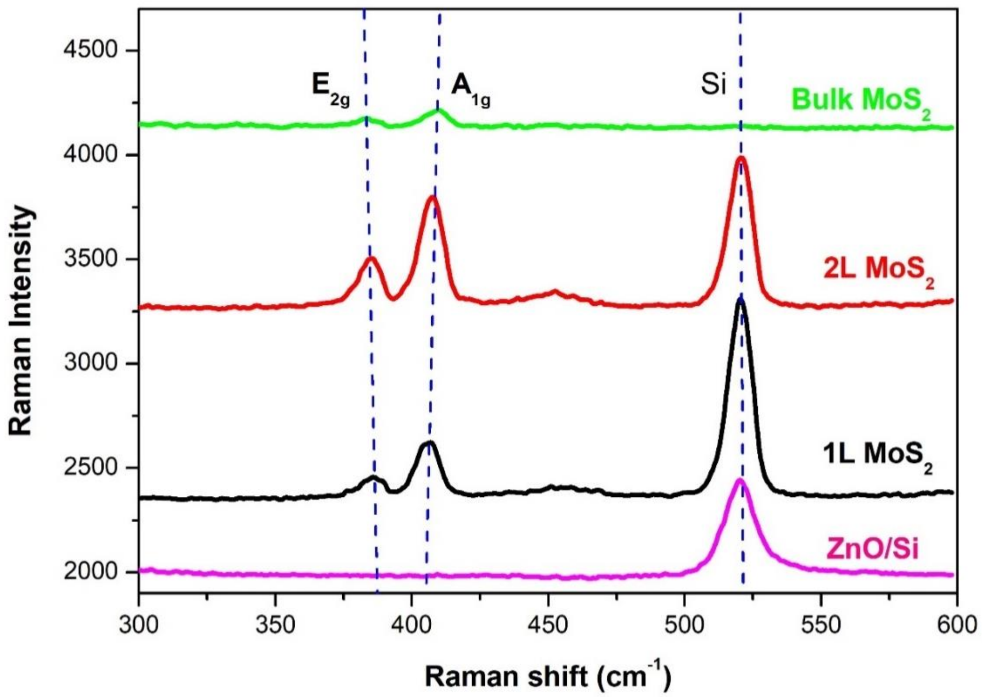

Supplementary Figure 3. The Raman spectrum of MoS_2 sheet with different layers on ZnO/Si substrate.

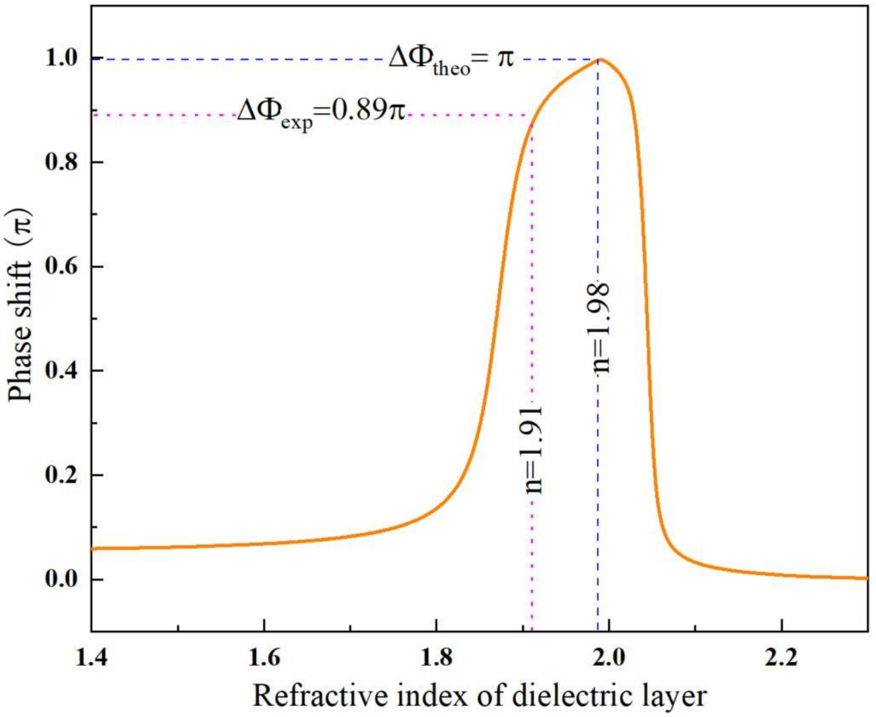

Supplementary Figure 4. Simulation results of the phase shift for a monolayer MoS_2 sheet on 65 nm thick dielectric layer with different refractive indices. The phase shift value will deviate from the peak value of π to 0.89 π when the refractive index of dielectric layer decrease from 1.98 to 1.91, which could explain the deviation of the phase shift measurement.

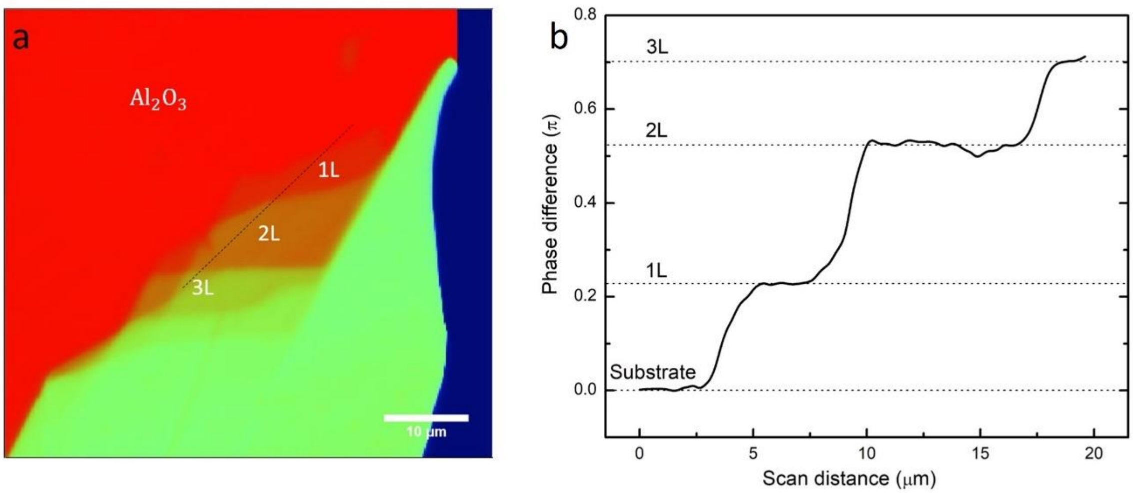

Supplementary Figure 5. (a) Phase shifting interferometry (PSI) image of a mechanically exfoliated MoS_2 flake on Al_2O_3/Si substrate, the thickness of Al_2O_3 layer is in its optimized value of 78 nm, the measured refractive index *n* is 1.65 at the wavelength of 535 nm. Different colors correspond to different thicknesses of MoS_2 flakes. The areas labeled as ‘‘1L’’, ‘‘2L’’ and ‘‘3L’’are single, bi- and tri-layer MoS_2, respectively. (b) PSI measured phase difference versus position of 1L, 2L and 3L MoS_2 along the dashed line marked in Supplementary Fig. 3a, which depicts that 0.22π, 0.53π and 0.7π phase shift have been achieved for single-, bi- and tri-layer MoS_2 sheet on Al_2O_3/Si substrate, respectively.

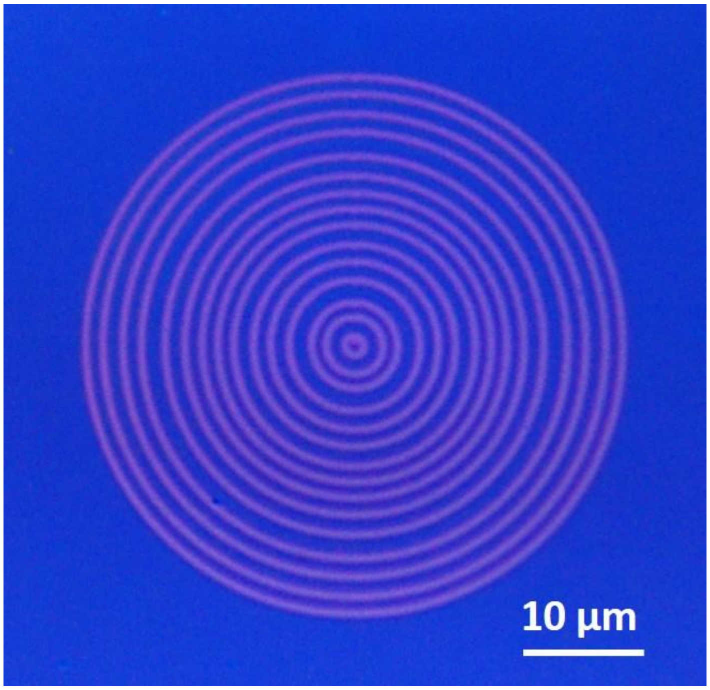

Supplementary Figure 6. Optical image of the laser scribed supercritical lens on a monolayer MoS_2 sheet

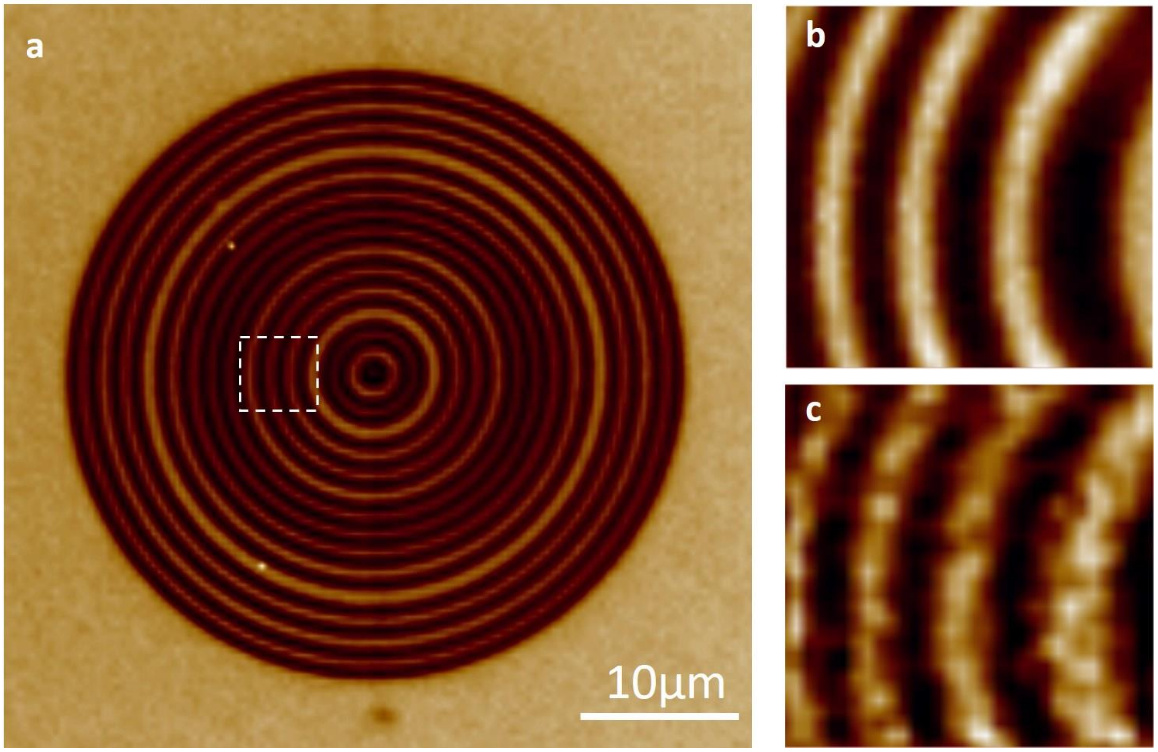

Supplementary Figure 7. (a) Raman mapping images of the supercritical lens on monolayer MoS_2 sheet. (b-c) The sectional mapping image of the dashed box region in (a) by plotting the integrated MoS_2 Raman A_{1g} peak intensity (b) and the Si Raman peak intensity (c) in confocal measurements.

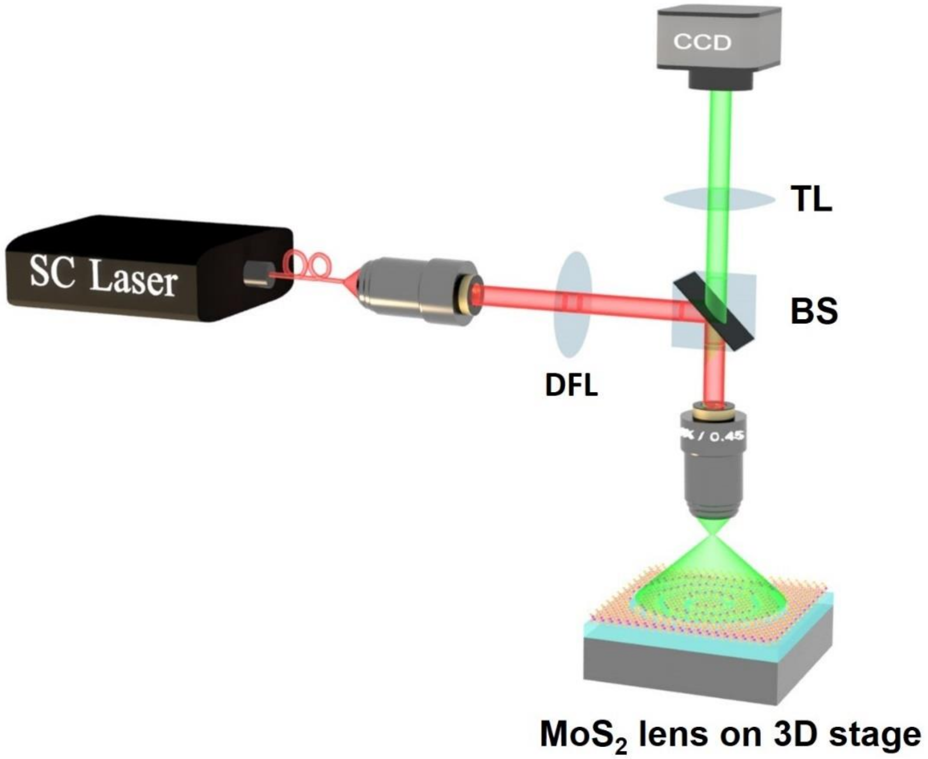

Supplementary Figure 8. The customized characterization system. Red light represents the illumination beam, and the Green light stands for the collected signal after modulation by the atomic thin supercritical lens; DFL: defocus lens; BS: beamsplitter; TL: Tube Lens.

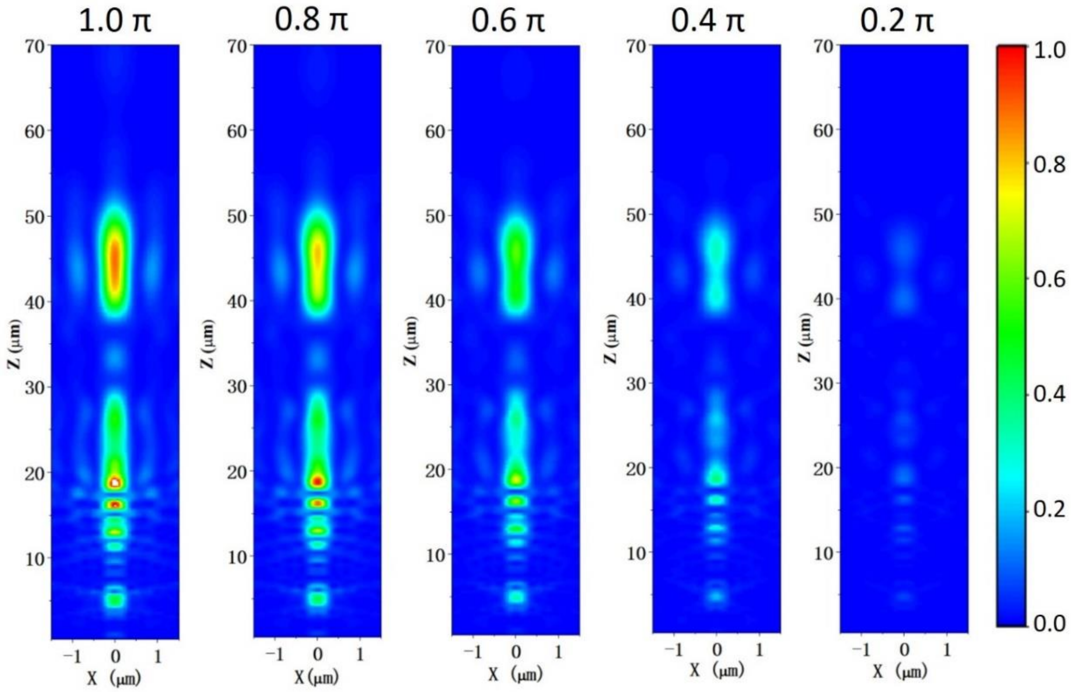

Supplementary Figure 9. Simulation results of XZ planar intensity distribution of the atomic thin SCL at the wavelength of 535 nm. When the phase shift changes from π to 0.2π, the focusing effect becomes weaker because the weak light modulation. But the position and lateral size of the focal spot remain unchanged.

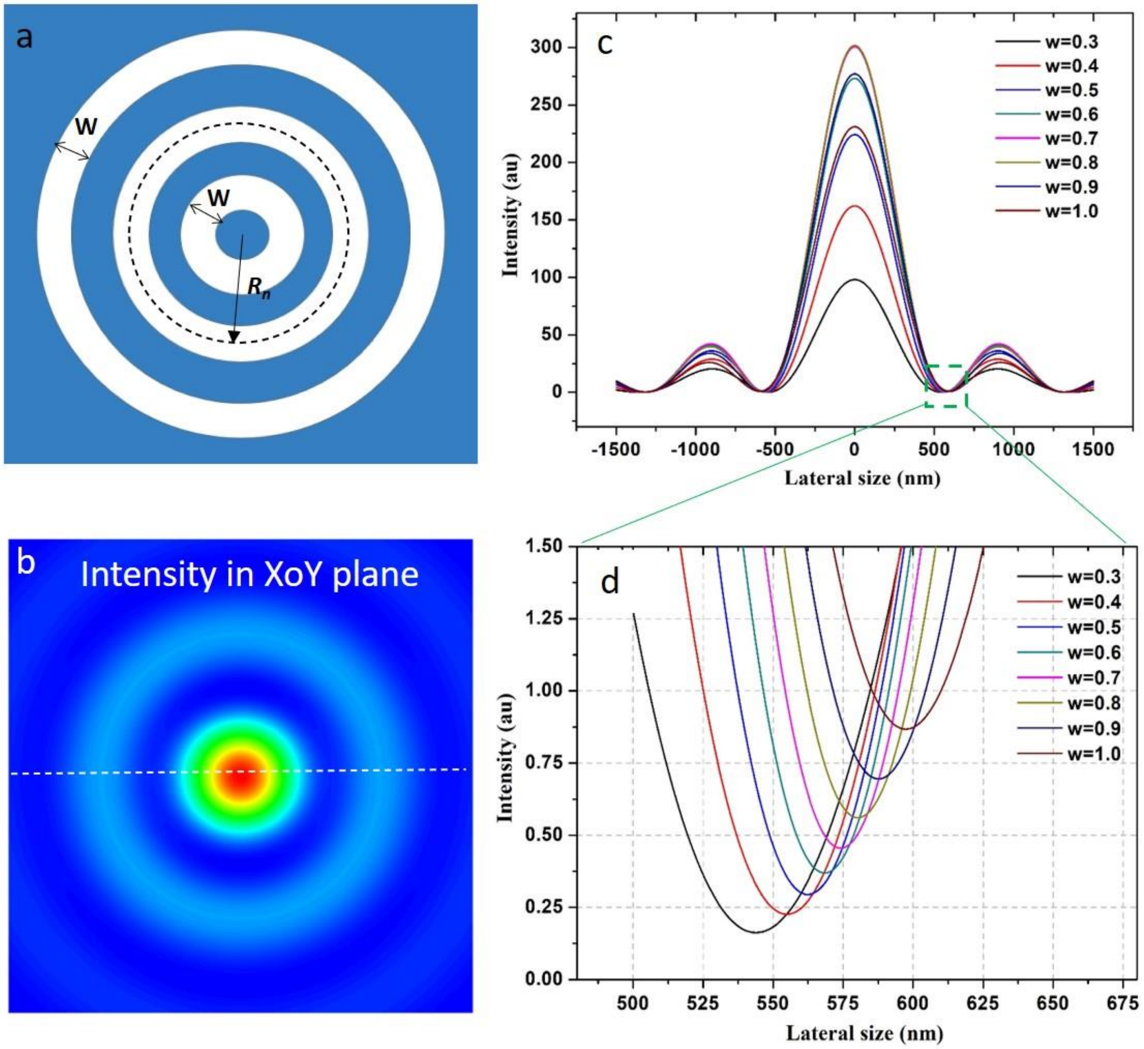

Supplementary Figure 10. The influence of the slot width (*w*) on the lateral size of sub-diffraction limited focal spot at the wavelength of 535 nm. (a) Schematic of the binary phase supercritical lens with fixed belt width of the laser scribed region. (b) Simulation results of the field distribution at the focal plane. (c) Line profile of the lateral intensity distribution for different belt widths, which show that the focusing intensity will gradually decrease with the belt width. (d) Zoom-in view of the dash box region in (c) indicates that binary phase supercritical lens with narrow scribed belts will get smaller lateral size of the focal spot. Overall consideration from the simulation result, $w = 0.5\mu m$ is the balance value between the spot intensity and spot size. Therefore, $w = 0.5$ μm is selected in our fabrication process.

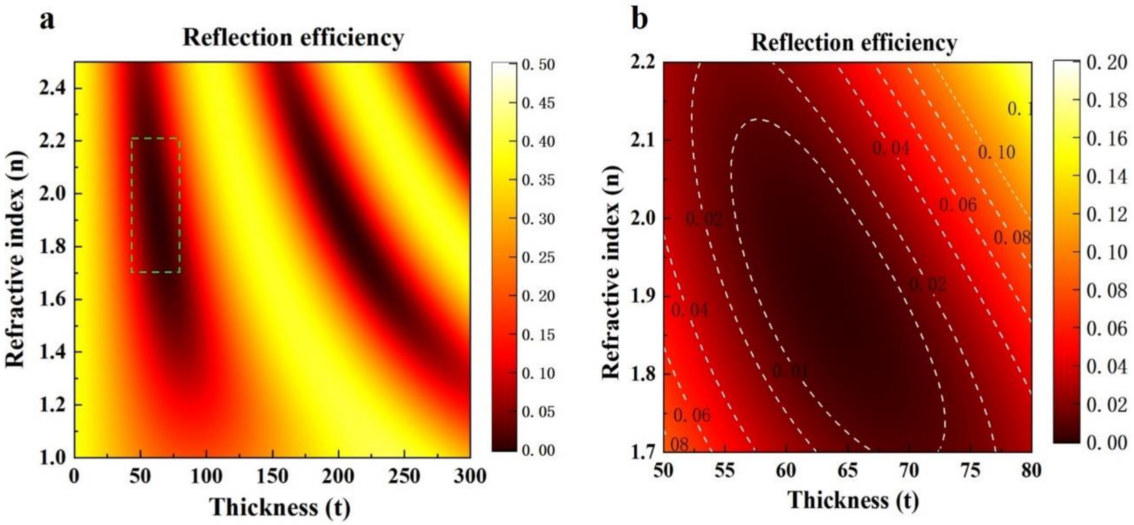

Supplementary Figure 11. (a) Simulation results of the reflection efficiency dependence on the thickness (*t*) and refractive index (*n*) of the dielectric layer. (b) Zoom-in view of the green dashed region in (a). White dashed lines are the iso-reflection contours.

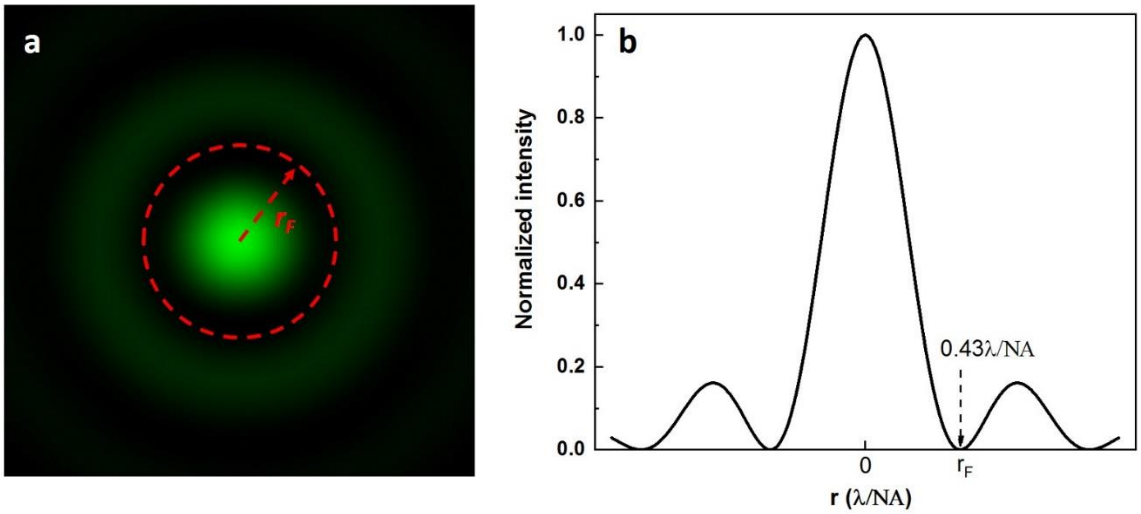

Supplementary Figure 12. Schematic shown of the focal spot region in the calculation of focusing efficiency. (a) The intensity distribution of the sub-diffractive limit focal spot at wavelength of 535nm, the area inside the red dashed circle represent the region of central hotspot, which adopted in numerical integrating about focusing efficiency. (b) Line profile of the lateral intensity distribution of the focal spot.

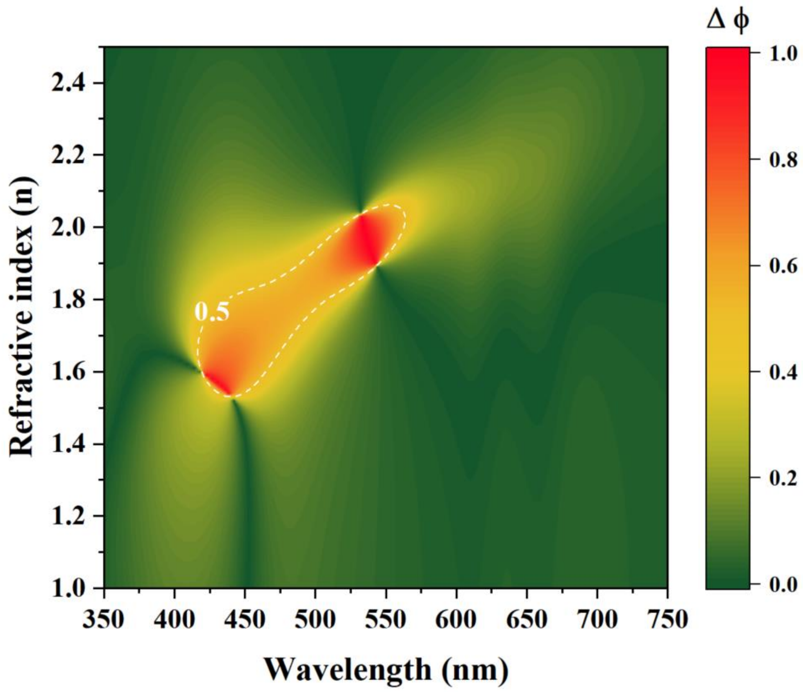

Supplementary Figure 13. Simulation results of the phase shift in broadband on the monolayer MoS_2 sheet with optimized dielectric layer thickness of 65 nm. The area inside the white dashed line is the region with phase shifts of 0.5π and above.

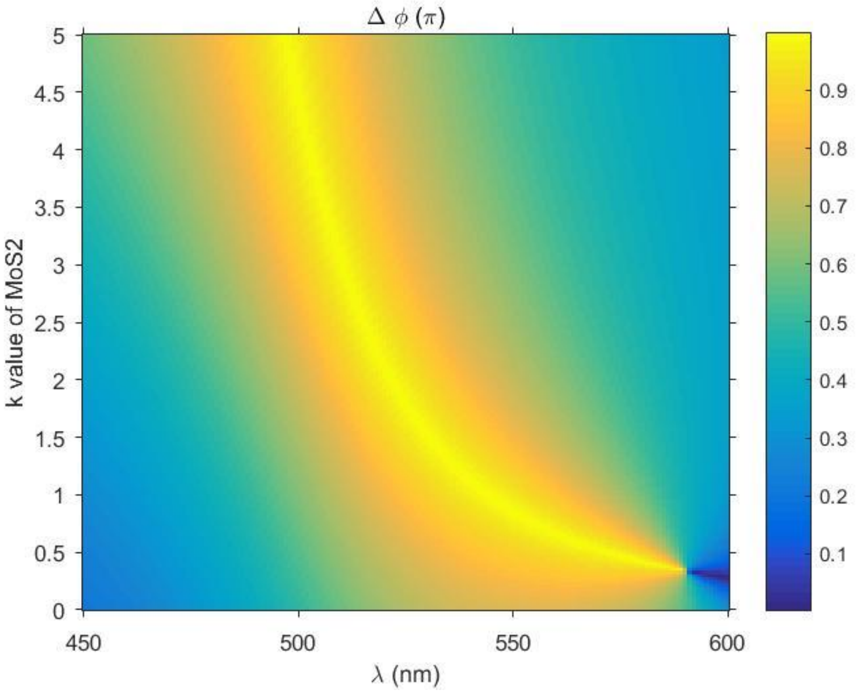

Supplementary Figure 14. The influence of imaginary part of refractive index of MoS_2 sheets on the loss-assisted Heaviside phase jump. The resonance wavelength with π phase shift will be shifted to longer wavelength along with the decreasing of the imaginary part of MoS_2 refractive index. Coincidentally, the *k* value of MoS_2 sheet is in the same trend in the wavelength range from 450 nm to 580 nm, which holds the key for broadband response.

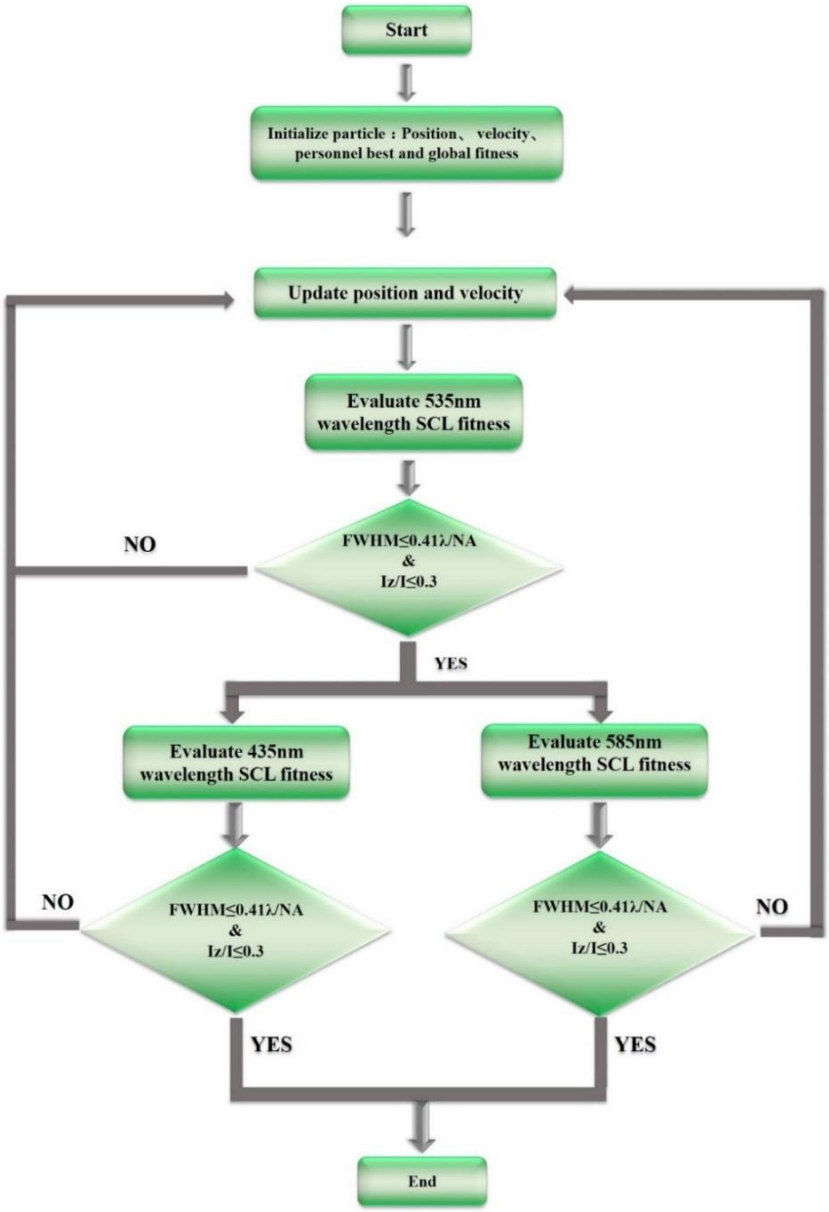

Supplementary Figure 15. The optimization procedure for broadband supercritical lens.

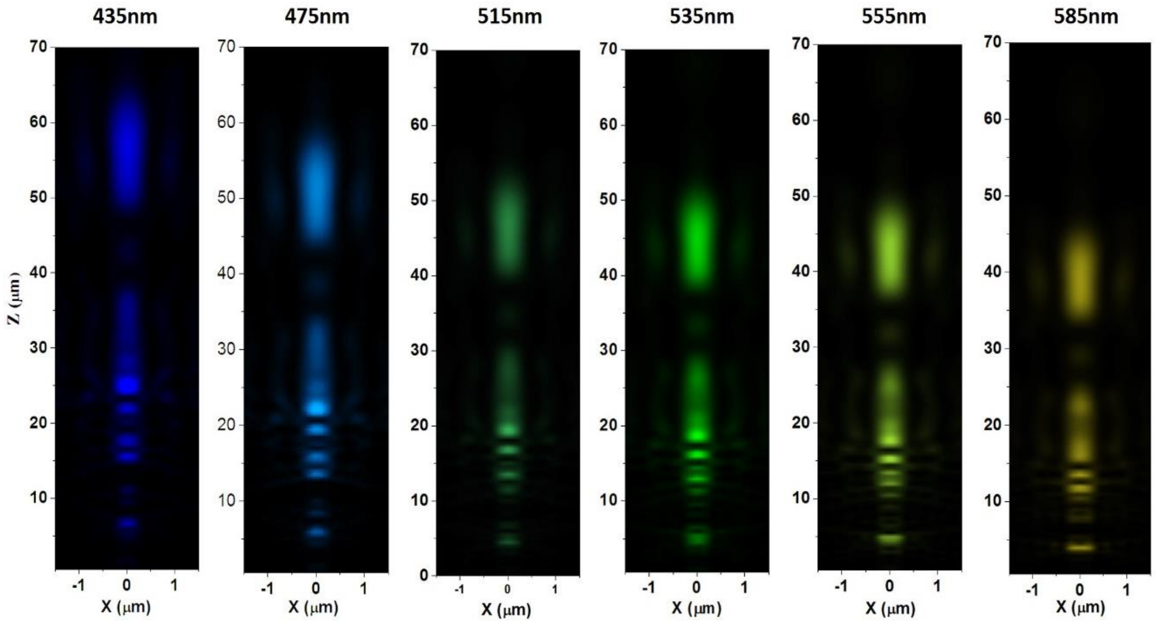

Supplementary Figure 16. Simulated intensity profile of the reflective beam by the atomic thin supercritical lens in the XZ plane at selected wavelengths from 435 nm to 585 nm.

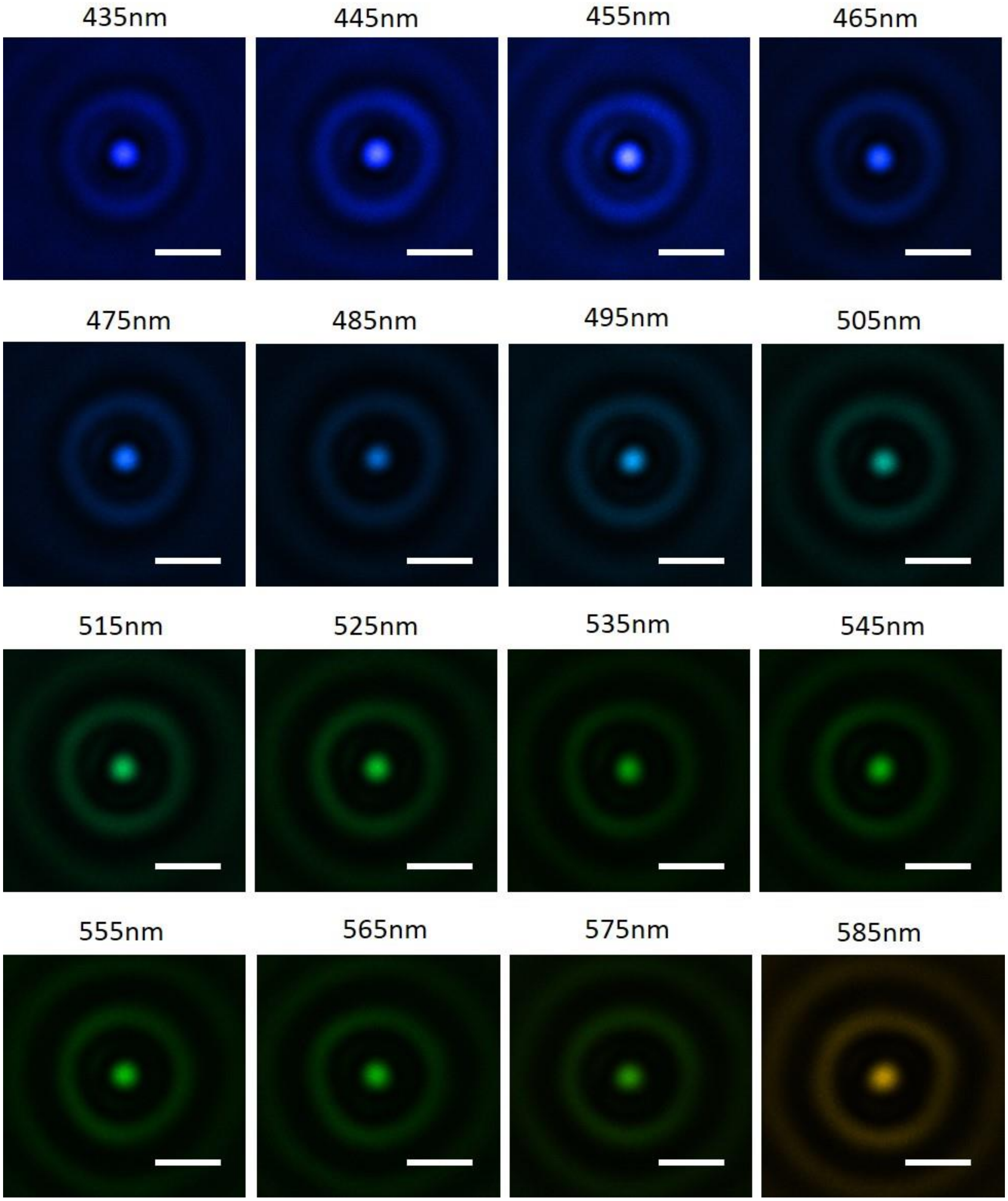

Supplementary Figure 17. Measured broadband focusing property of the atomic thin supercritical lens in the wavelength range from 435 nm to 585 nm. Scale bar: 1000nm.

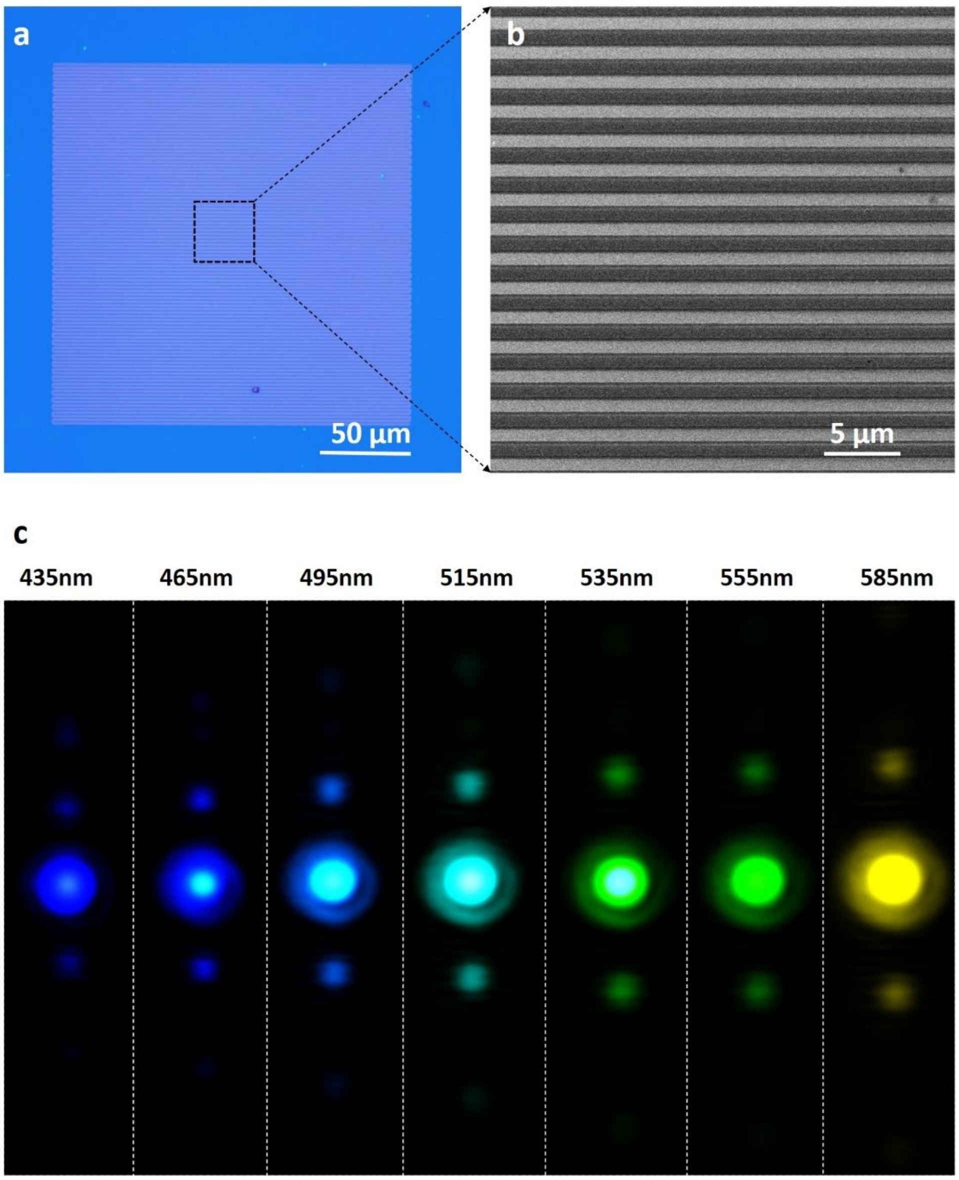

Supplementary Figure 18. (a) Optical microscopy image of an atomic thin grating on bilayer MoS_2 sheet. The size of the MoS_2 grating is 200μm × 200 μm. Pitch of the grating is 2 μm. (b) SEM image of sectional zoom-in view for the central black dash line region in (a). (c) Broadband beam deflection capability by a MoS_2 grating.

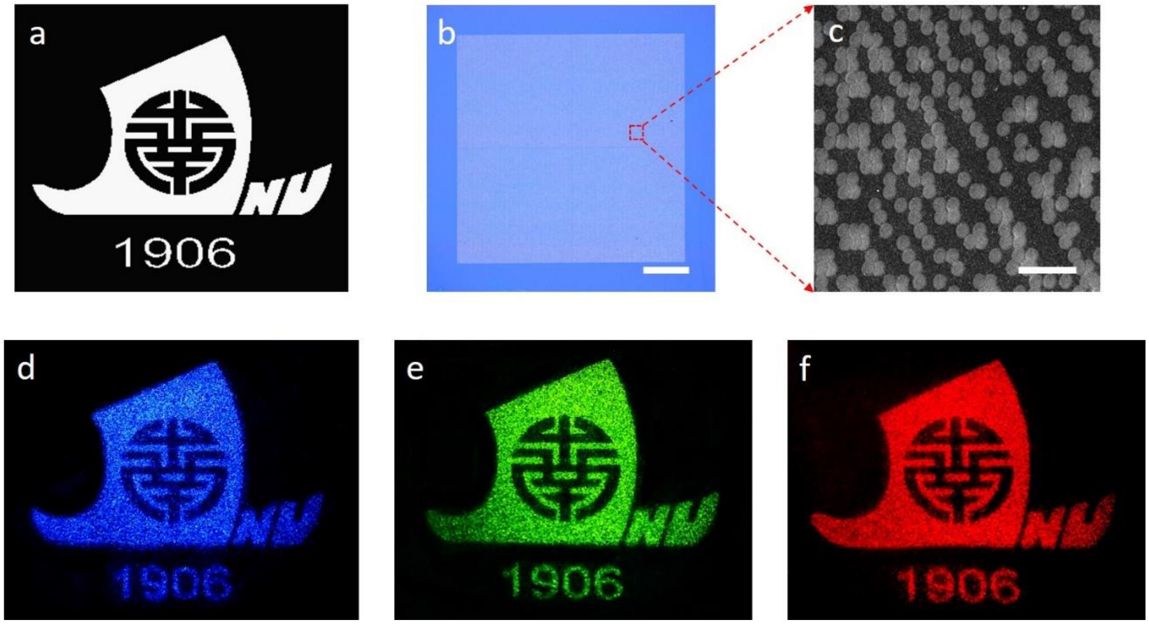

Supplementary Figure 19. Demonstration of meta-holograms on atomically-thin MoS_2 sheet. (a) Target image for the holographic demonstration. (b) Optical images of pixel array patterned by fs laser scribing. Scale bar: 200 μm. (c) Zoom-in view SEM image of the selective region marked by red dashed square in (b). The bright spots stand for the scribed region by fs laser. Scale bar: 5 μm. (d-f) Holographic images captured at the illumination wavelengths of 473 nm, 561 nm and 633 nm, respectively.

Supplementary Reference:

1. Saleh, B.E.A. & Teich, M.C. Fundamentals of photonics, Vol. 22. (John Wiley & Sons, Inc., New York; 1991).
2. Yang, J. et al. Atomically thin optical lenses and gratings. *Light Sci. Appl.* **5**, e16046 (2016).
3. Liu, H.-L. et al. Optical properties of monolayer transition metal dichalcogenides probed by spectroscopic ellipsometry. *Appl. Phys. Lett.* **105**, 201905 (2014).
4. Palik, E.D. Handbook of Optical Constants of Solids. (Academic Press, San Diego, 1998).
5. Ji, J. et al. Giant magneto-optical Raman effect in a layered transition metal compound. *Proc. Natl. Acad. Sci.* **113**, 2349-2353 (2016).
6. Tapily, K., Gu, D., Baumgart, H., Rigo, M. & Seo, J. Raman Spectroscopy of ZnO Thin Films by Atomic Layer Deposition. *ECS Trans.* **33**, 117-123 (2010).
7. Qin, F. et al. A Supercritical Lens Optical Label-Free Microscopy: Sub-Diffraction Resolution and Ultra-Long Working Distance. *Adv. Mater.* **29**, 1602721 (2017).
8. Huang, K. et al. Planar Diffractive Lenses: Fundamentals, Functionalities, and Applications. *Adv. Mater.* **30**, e1704556 (2018).
9. Huang, K. et al. Optimization-free superoscillatory lens using phase and amplitude masks. *Laser Photon. Rev.* **8**, 152-157 (2014).
10. Yuan, G., Rogers, E.T. & Zheludev, N.I. Achromatic super-oscillatory lenses with sub-wavelength focusing. *Light Sci. Appl.* **6**, e17036 (2017).
11. Tang, D. et al. Ultrabroadband superoscillatory lens composed by plasmonic metasurfaces for subdiffraction light focusing. *Laser Photon. Rev.* **9**, 713-719 (2015).
